# COVID-19 in children: Clinical presentation and hospital course at a district hospital in South Africa

**DOI:** 10.4102/sajid.v39i1.580

**Published:** 2024-07-08

**Authors:** Mareli Nieuwoudt, Natasha L. O’Connell, Marieke M. van der Zalm, Andrew W. Redfern, Helena Rabie

**Affiliations:** 1 Department of Paediatrics and Child Health, Faculty of Medicine and Health Sciences, Stellenbosch University, Cape Town, South Africa; 2 Department of Paediatrics and Child Health, Khayelitsha Hospital, Cape Town, South Africa

**Keywords:** COVID-19, infant, child, Africa, LMICs

## Abstract

Limited data exist on South African children hospitalised with COVID-19 in district hospitals. We describe the presentation and outcomes of children admitted to a level 1 and 2 hospital and compare this with children admitted to a level 2 and 3 hospital.

**Contribution:**

This study highlights that young age is an important risk factor for hospitalisation with severe COVID-19. Infants with HIV exposure and prematurity are disproportionately represented among admissions. Furthermore, we notice a high number of children with current or new tuberculosis confirming the interplay between viral infections and childhood tuberculosis.

## Introduction

Pneumonia is a major cause of child mortality globally with higher rates of death in sub-Saharan Africa.^1^ Unlike the severe outcomes of adults with severe acute respiratory syndrome coronavirus-2 (SARS-CoV-2), children typically have a milder form of the SARS-CoV-2 disease unless they are very young or have pre-existing conditions.^2^ The most common presenting symptoms in children are fever and cough. Other less common presenting complaints include rhinorrhoea, sore throat, diarrhoea, and vomiting.^3,4^

Limited paediatric data from settings with high rates of poverty, HIV, tuberculosis, and malnutrition make it challenging to comprehend the diverse impacts of SARS-CoV-2 in children across different income levels. However, current research indicates that COVID-19 morbidity and mortality are more pronounced in children in low- and middle-income countries (LMICs) compared to their counterparts in high-income countries.^5,6^

Current data of South African children with severe COVID-19 or MIS-C mostly derive from level 3 hospitals and may not represent the entire spectrum of disease in hospitalised children.^3^

This study focuses on hospitalised children with COVID-19 at a district hospital and a central hospital, which are identified in this research article as DH (for district hospital) and CH (for central hospital). The district hospital (DH) is in the Cape Town Metro area with some level 2 services excluding paediatric high care or intensive care. The DH refers some of their cases to the CH. The CH is a level 2 and 3 hospital. We compare paediatric COVID-19 cases and outcomes admitted to the DH with those admitted to the CH.

## Research methods and design

We reviewed the DH admission register and identified children aged 0–13 who had a positive real-time polymerase chain reaction (RT-PCR) for SARS-CoV-2 on a respiratory specimen and were admitted to the paediatric ward at DH between April 01st and September 30th, 2020. Respiratory samples included nasopharyngeal aspirates, tracheal aspirates, or nasopharyngeal swabs. Severe acute respiratory syndrome coronavirus-2 rapid antigen tests were not used during the study period.

Clinical data including pre-existing conditions, presentation, interventions required, and progress were extracted from the medical and laboratory records. Children from CH admitted with positive RT-PCR over the same period were included in a prior study describing presentation and outcomes.^3^ Paediatric patients transferred from DH to CH were included in the DH cohort and were subsequently excluded from the CH dataset. Infants admitted to neonatal wards were excluded from the study. Prematurity was considered a pre-existing medical condition if the age at presentation was ≤ 12 months. Similarly, HIV exposure was only considered if the child was ≤ 18 months old.

In the study period, DH and CH tested children meeting the case criteria for possible COVID-19. As the epidemic was developing, CH additionally tested all suspected MIS-C cases and all intensive care admissions from May 2020, as well as all children requiring procedural intubation from June 2020. At DH outpatients were tested up to May 2020 but thereafter only inpatients where tested. Need for isolation was not an admission criterion at either DH or CH.

Deidentified data were entered into a database and analysed using SPSS Inc. (2001 Chicago United States version 26). Descriptive statistics were used for demographic and clinical characteristics. Categorical variables were compared using the Chi-squared or Fisher exact test, and continuous variables were analysed using the Mann–Whitney U test in the univariate analysis. Statistical significance was defined as a *p*-value less than 0.05.

Weight-for-age *z*-scores were calculated using World Health Organization growth reference data with Anthro and Anthroplus software. Weight-for-age percentiles for children above 10 years were determined using Centers for Disease Control and Prevention Growth Charts (2000) via AnthroCalc Application version 2.3.^7^

### Ethical considerations

Ethical clearance to conduct this study was obtained from the Human Research Ethic Committee of the Faculty of Health Sciences, Stellenbosch University, South Africa (HREC N20/04/013_COVID).

All procedures performed in studies involving human participants were in accordance with the ethical standards of the institutional and/or national research committee and with the 1964 Helsinki Declaration and its later amendments or comparable ethical standards.

The data were entered without patient identifiers using only routinely collected data. A waiver of consent for this process was obtained (HREC N20/04/013_COVID).

## Results

In the study period, 126 children with positive SARS-CoV-2-PCR tests were hospitalised, 88 (69.8%) at CH and 38 (30.2%) at DH. There was a slight male predominance, 71/126 (56.3%) (Table 2a and Table 2b). The median age of the overall group was 13 months (Interquartile range 2.0–43.5). Sixty-two (49%) were younger than 12 months of age. Pre-existing conditions were present in 73/126 (57.9%) patients; 15 of 60 children with known information had a history of prematurity. Twenty-five of the 64 children < 18 months (39%) with available information were HIV-exposed. Nine patients (9/119, 7.6%) had a current or new diagnosis of pulmonary TB (Table 2a and Table 2b).

### Clinical presentation of children at the District Hospital (DH)

The median age during admission at DH was 8.5 months (IQR2.0–31.0) and 20/38 (52.6%) were younger than 12 months of age (Table 2a and Table 2b). Pre-existing conditions were present in 18/38 (47.4%) and most frequently included prematurity (15.8%), epilepsy (15.8%), tuberculosis (10.5%), and cardiac disease (7.9%). Half of the patients less than 18 months old were HIV exposed but uninfected (11/22, 50%). Only one patient at DH was living with HIV (1/36, 2.8%) (Table 1a and Table 1b).

**TABLE 1a:** Characteristics of children with positive severe acute respiratory syndrome coronavirus-2 real-time polymerase chain reaction hospitalised at the District Hospital by age.

Characteristic	Children infected (*N* = 38)		0 – 3 months (*N* = 13)		3 months – 12 months (*N* = 7)		12 months – 60 months (*N* = 12)		> 5 years (*N* = 6)		*p* *
	*n*	%	*n*	%	*n*	%	*n*	%	*n*	%	
Gender, male	26	68.4	10	76.9	3	42.9	9	75.0	4	66.7	0.42
BCG vaccination history or scar	34¶	97.1	12§§	100	7	100	10¶¶	90.9	5‡‡‡	100	0.52
HIV infection	1††	2.8	0§§	0	0	-	1¶¶	9.1	0	-	0.50
HIV exposed uninfected	14††	38.9	6	46.2	3	42.9	5¶¶	45.5	0‡‡‡	-	0.29
Premature	5‡‡	15.6	2§§	16.7	1	14.3	2†††	22.2	0§§§	-	0.79
Haematology	1	2.6	1	7.7	0	-	0	-	0	-	0.58
Malignancy	1	2.6	0	-	0	-	0	-	1	16.7	0.14
**Clinical presentation**											
Respiratory	26	68.4	10	76.9	6	85.7	7	58.3	3	50.0	-
URTI†	2	5.3	1	7.7	0	-	1	8.3	0	-	-
LRTI	25	65.8	10	76.9	6	85.7	6	50.0	3	50.0	0.26
Gastrointestinal	8	21.1	3	23.1	2	28.6	2	16.7	1	16.7	-
Infectious‡	8	21.1	3	23.1	1	14.3	4	33.3	0	-	-
Neurology§	5	13.2	0	-	0	-	2	16.7	3	-	-
Other‡	7	18.4	2	15.4	2	28.6	2	16.7	1	16.7	-
Any oxygen support	25	65.7	8	61.5	6	85.7	8	66.7	3	50.0	0.57
Low flow nasal cannula oxygen	22	57.9	8	61.5	6	85.7	6	50	2	33.3	0.25
High flow nasal cannula oxygen	1	2.6	0	-	0	-	1	8.3	0	-	0.78
Continuous positive airway pressure	0	-	0	-	0	-	0	-	0	-	-
Intubated for transfer	2	5.3	0	-	0	-	1	8.3	1	16.7	0.04
**Outcome**											
COVID-related death	1	2.6	1	7.7	0	-	0	-	0	-	0.58
Transfer	9	23.7	3	23.1	0	-	3	25	3	50	0.21
PICU admission	2	5.3	0	-	0	-	1	8.3	1	16.7	0.04

*Source:* Van der Zalm MM, Lishman J, Verhagen LM, et al. Clinical experience with severe acute respiratory syndrome coronavirus 2–related illness in children: Hospital experience in Cape Town, South Africa. Clin Infect Dis. 2021;72(12):938–944. https://doi.org/10.1093/cid/ciaa1666

LRTI, lower respiratory tract infections; URTI, upper respiratory tract infection; PICU, paediatric intensive care unit; BCG, bacille Calmette-Guerin vaccine.

* , *p*-values were calculated using the Chi-squared/Fisher exact test for categorical variables, and the Mann–Whitney U test for continuous variables.

† , Of the two children diagnosed with URTI: One had associated acute gastroenteritis and one had associated pneumonia;

‡ , Presumed sepsis in infants < 3 months, meningitis, and tuberculosis;

§ , Included: Breakthrough seizures in patients with epilepsy x 5;

¶ , *N* = 35;

†† , *N* = 36;

‡‡ , *N* = 32;

§§ , *N* = 12;

¶¶ , *N* = 11;

††† , *N* = 9;

‡‡‡ , *N* = 5;

§§§ , *N* = 4.

**TABLE 1b:** Characteristics of children with positive severe acute respiratory syndrome coronavirus-2 real-time polymerase chain reaction hospitalised at the District Hospital by age.

Characteristic	All children *N* = 38		0 – 3 months *N* = 13		3 months – 12 months *N* = 7		12 months – 60 months *N* = 12		> 5 years *N* = 6		*p* *
	Median	IQR	Median	IQR	Median	IQR	Median	IQR	Median	IQR	
WAZ, z score -WHO ≤ 10 years)†‡§	−0.14	−1.48 to 0.69	−1.10	−1.9 to - 0.1	0.56	−1.85 to 1.07	−0.20	−0.86 to 0.65	1.36	0.36 to 3.10	0.37
**Outcome**											
Length of stay (days)	4.5	3.0-11.0	4.0	3.0-7.0	4.0	2.0-5.0	4.5	3.0-10.5	15.0	8.0-19.0	0.38

*Source:* Van der Zalm MM, Lishman J, Verhagen LM, et al. Clinical experience with severe acute respiratory syndrome coronavirus 2–related illness in children: Hospital experience in Cape Town, South Africa. Clin Infect Dis. 2021;72(12):938–944. https://doi.org/10.1093/cid/ciaa1666

CDC, Centers for Disease Control and Prevention; WAZ, weight-for-age z score; IQR, interquartile range.

* , *p*-values were calculated using the Chi-squared/Fisher exact test for categorical variables, and the Mann–Whitney U test for continuous variables.

† , For children older than 10 years we used the Centers for Disease Control and Prevention (CDC) weight for age calculation in percentiles see text (*n* = 2; 84.5%, 65.5%);

‡ , weight-for- age z score (WAZ) was less than –2 for five patients (14.3%);

§ , *N* = 35.

The majority (25/38, 65.8%) of admissions at DH had clinical features suggesting lower respiratory tract infections (LRTIs), necessitating oxygen therapy in 91.7%. No significant difference was noticed in the need for supplemental oxygen between the different age groups (*p* = 0.57). Diarrhoea and vomiting were more frequent in children ≤ 12 months when compared to older children (5/20, 25.0% vs. 3/18, 16.7%). Seizures were observed in five children (5/38, 13.2%); four of these children had normal cerebrospinal fluid analysis and a lumbar puncture was not performed in one child because of high acuity (Table 1a and Table 1b).

Nine children (9/38, 23.7%) required a transfer to a level-3 hospital. The reasons for transfer included sub-specialty care (4/9, 44.4%) and admission to the Paediatric Intensive Care Unit and/or need for continuous positive airway pressure (CPAP) or high-flow nasal cannula oxygen (HFNCO_2_) (5/9, 55.6%). Among patients transferred for respiratory support, 60% continued CPAP or HFNCO_2_, while 40% received nasal prong oxygen at CH. Sub-specialty care transfers included cases of suspected haematological malignancy (oncology), autoimmune encephalitis (neurology), difficulty in weaning supplemental oxygen with suspected drug-resistant TB, and one patient with concern for TB mediastinal lymph nodes causing airway compression (pulmonology). Four of the transferred patients received new TB diagnoses (4/9, 44.4%).

The median duration of DH stay was 4.5 days (IQR 3.0–11.0). A single fatality at DH was attributed to COVID-19. This 2-month-old infant was deceased upon arrival and had a posthumous diagnosis of pneumonia based on chest X-ray and a SARS-CoV-2 PCR positive respiratory sample. (Table 1a and Table 1b).

### Comparison with the Central Hospital

Children admitted at CH were slightly older compared to those at DH, with a median age of 14.0 months (IQR 2.0–50.0, *p* = 0.54) (Table 2a and Table 2b). Almost half of the children were 12 months of age and younger in both cohorts (20/38, 52.6% at DH and 42/88, 47.8% at CH, *p* = 0.90) (Table 2a and Table 2b).

**TABLE 2a:** Comparing the characteristics of children with positive severe acute respiratory syndrome coronavirus-2 real-time polymerase chain reaction at the District Hospital to those at the Central Hospital.

Characteristic	All children (*N* = 126)		CH (*N* = 88)		DH (*N* = 38)		*p*
	*n*	%	*n*	%	*n*	%	
Gender (male)	71	56.3	45	51.1	26	68.4	0.07
**Age**							0.84
0 to ≤ 3 months	41	32.5	28	31.8	13	34.2	-
> 3 to ≤ 12 months	21	16.7	14	15.9	7	18.4	-
> 12 months to ≤ 60 months	39	31.0	27	30.7	12	31.6	-
> 5 years	25	19.8	19	21.6	6	15.8	-
**Any Comorbidities** †	73	57.9	55	62.5	18	47.4	0.11
Prematurity‡	15¶¶	25.0	12	29.3	3‡‡‡‡‡	15.8	-
Hypertension	1	0.8	0	-	1	2.6	-
Previous tuberculosis	2†††	1.8	2‡‡‡‡	2.6	0	-	-
Current tuberculosis	9‡‡‡	7.6	5	6.2	4	10.5	-
Sickle cell anaemia	3	2.4	2	2.3	1	2.6	-
Malignancy	8	6.3	1	8.0	1	2.6	-
Chronic kidney disease	3	2.4	3	3.4	0	-	-
Chronic neurological disease§	3	2.4	1	1.1	2	5.3	-
Epilepsy	8	6.3	2	2.3	6	15.8	-
HIV infection	3§§§	2.9	2§§§§	2.9	1§§§§§	2.8	-
Cardiac disease	11	8.7	8	9.1	3	7.9	-
Asthma	2	1.6	1	1.1	1	2.6	-
Chronic lung disease	4	3.2	3	3.4	1	2.6	-
Cerebral Palsy	3	2.4	0	-	3	7.9	-
Other¶	15	11.9	15	17.0	0	-	-
HIV exposed††	25¶¶¶	39.1	14¶¶¶¶	33.3	11¶¶¶¶¶	50.0	0.19
**Symptoms**							-
Cough	45	35.7	26	29.5	19	50	0.03
Fever	24	19	15	17.0	9	23.7	0.38
Tight chest	33	26.2	19	21.6	14	36.8	0.07
**Gastrointestinal tract symptoms**							
Abdominal pain	13	10.3	13	14.8	0	-	0.01
Diarrhoea	21	16.7	11	12.5	10	26.3	0.05
Vomiting	38	30.2	24	27.3	14	36.8	0.28
Convulsions	11	8.7	6	6.8	5	13.2	0.25
Rash‡‡	14	11.1	9	10.2	5	13.2	0.63
**Outcomes**							
Supplemental oxygen§§	62††††	50.8	37†††††	44.0	25	65.8	0.03
COVID-19 related death	2	1.6	1	1.1	1	2.6	0.78

*Source*: Van der Zalm MM, Lishman J, Verhagen LM, et al. Clinical experience with severe acute respiratory syndrome coronavirus 2–related illness in children: Hospital experience in Cape Town, South Africa. Clin Infect Dis. 2021;72(12):938–944. https://doi.org/10.1093/cid/ciaa1666

DH, District Hospital; CH, Central Hospital.

† , More than one comorbidity was present in 16 children;

‡ , Included babies born before 37 completed weeks gestational age; only children ≤ 12 months were included;

§ , Excluded: Epilepsy and cerebral palsy;

¶ , Included: Systemic autoimmune diseases × 2, Genetic conditions × 2, Foetal alcohol syndrome × 1, Newly diagnosed type 1 diabetes × 1, Severe acute malnutrition × 2, Hirschsprung’s disease × 1, Rickets × 2, Osteogenesis imperfecta × 1, Congenital vocal cord palsy × 1, Pure red cell aplasia × 1, Severe idiopathic aplastic anaemia × 1;

†† , Only children younger than 18 months were included.

‡‡ , At DH included: Generalised macular rash on body, face and limbs with oedematous hands and feet × 1, chemical dermatitis × 1, details not noted × 1, lower limb macular erythematous pruritic rash × 1, resolving impetigo × 1;

§§ , Includes any form of supplementary oxygen administration;

¶¶ , *N* = 60;

††† , *N* = 114;

‡‡‡ , *N* = 119;

§§§ , *N* = 105;

¶¶¶ , *N* = 64;

†††† , *N* = 122;

‡‡‡‡ , *N* = 76;

§§§§ , *N* = 69;

¶¶¶¶ *N* = 42;

††††† , *N* = 84;

‡‡‡‡‡ , *N* = 17;

§§§§§ , *N* = 36;

¶¶¶¶¶ , *N* = 22.

**TABLE 2b:** Comparing the characteristics of children with positive severe acute respiratory syndrome coronavirus-2 real-time polymerase chain reaction at the District Hospital to those at the Central Hospital.

Characteristic	All children (*N* = 126)		CH (*N* = 88)		DH (*N* = 38)		*p*
	Median	IQR	Median	IQR	Median	IQR	
Age (months)	13	2.00— 43.5	14.0	2.0—50.0	8.5	2.0—31.0	0.54
**Outcomes**							
Length of stay (days)	5.0	2.5—13.0	5.6	2.0—13.5	4.5	3.0—11.0	0.56

DH, District Hospital; CH, Central Hospital; IQR, interquartile range.

At DH children more often presented with coughing, chest tightness (19/38, 50.0% vs. 26/88, 29.5%, *p* = 0.03, 14/38, 36.8% vs 19/88, 21.6%, *p* = 0.07, respectively), and diarrhoea (10/38, 26.3% vs. 11/88, 12.5%, *p* = 0.05) compared to CH. Pre-existing conditions were slightly more common at CH (55/88, 62.5%) than DH (18/38, 47.4%, *p* = 0.11). Perinatal HIV exposure was frequent in both cohorts (DH: 11/22, 50.0%, CH: 14/42, 33.3%, *p* = 0.19), although HIV infection rates were low (DH 1/36, 2.8 % vs CH 2/69, 2.9%). Tuberculosis diagnoses were comparable between DH (4/38, 10.5%) and CH (5/81, 6.2%, *p* = 0.40). None of the children with TB were known to be living with HIV. Similar median hospital admission durations (DH: 4.5 days, IQR 3.0–11.0, CH: 5.6 days, IQR 2.0–13.5, *p* = 0.56) were noticed. A clinically significant difference was observed between the oxygen requirements at DH (25/38, 65.8%) compared to that at CH (37/84, 44%, *p* = 0.03) (Table 2a and Table 2b).

## Discussion

Children managed at DH did not differ significantly from those reported in literature. They were young, mostly presenting with LRTIs requiring oxygen support and nearly half had pre-existing or newly diagnosed co-morbidities.^3,8^

Tuberculosis was diagnosed in 10.5% of children in the DH cohort, compared to 6.2% in CH. In adults, evidence suggests that COVID-19 and TB co-infection exacerbates the severity of COVID-19 and may contribute to the progression of TB. The shared immune responses between COVID-19 and tuberculosis suggest potential dual risks.^9,10^ The role of childhood TB in COVID-19 severity remains poorly understood, although current South African data focusing on Omicron SARS-COV-2 do not prove an increased risk of severe COVID-19 in children with TB or those living with HIV.^11^

HIV-exposed but uninfected children are disproportionately represented among those requiring hospitalisation because of SARS-CoV-2 in both cohorts. The rates of HIV exposure in this group are notably higher than the antenatal prevalence of HIV in the Western Cape.^4,12^ A study comparing acute respiratory illnesses in South African children found a higher SARS-CoV-2 infection risk in HIV-exposed but uninfected children.^13^ While suggesting a potential association between HIV exposure and severe LRTIs because of SARS-CoV-2, the heightened prevalence of HIV exposure and severity of SARS-CoV-2-induced LRTIs could not be fully explored in this small retrospective study. Increased risk of more severe LRTIs is well-documented in these infants.^14^

Clinical differences between DH and CH cases included age distribution, comorbidity prevalence, and gastrointestinal symptoms, yet both centres predominantly observed LRTIs necessitating oxygen support, with no disparity in hospital admission duration.

This study is limited by its retrospective nature including poorly documented oxygen saturation prior to initiation of supplementary oxygen. In addition, the sample size is small and all children were not routinely investigated for other viral infections. Variations in screening protocols between the two hospitals throughout the study period caused potential impact on the study outcomes. Furthermore, challenges were encountered in attributing COVID-19 to specific clinical syndromes in selected cases.

## Conclusion

Our findings underscore the severity of COVID-19 especially in infants, and children with comorbidities, emphasising the vital role of widespread oxygen availability, oxygen saturation monitoring, and oxygen support strategies at level 1 and 2 facilities to mitigate the impact of viral pneumonia in African countries.
